# Public support for smoke-free private indoor and public outdoor areas in the Netherlands: A trend analysis from 2018–2022

**DOI:** 10.18332/tid/176141

**Published:** 2024-01-17

**Authors:** Nienke W. Boderie, Sabri Ennissay, Wilhelmina Ijzelenberg, Frank J. van Lenthe, Jessica Baars, Jasper V. Been

**Affiliations:** 1 Department of Public Health, Erasmus MC Rotterdam, University Medical Center Rotterdam, Rotterdam, The Netherlands; 2 Amsterdam Public Health Research Institute, Department of Health Sciences, Faculty of Sciences, Vrije Universiteit Amsterdam, Amsterdam, The Netherlands; 3 Health Funds for a Smokefree Netherlands, Utrecht, the Netherlands; 4 Department of Neonatal and Paediatric Intensive Care, Division of Neonatology, Erasmus MC Sophia Children's Hospital, University Medical Center Rotterdam, Rotterdam, Netherlands; 5 Department of Obstetrics and Gynaecology, Erasmus MC Sophia Children's Hospital, University Medical Center Rotterdam, Rotterdam, Netherlands

**Keywords:** smoke-free zones, public support, repeated crosssectional

## Abstract

**INTRODUCTION:**

In addition to smoke-free policies in indoor public and workplaces, governments increasingly implement smoke-free policies at beaches, in parks, playgrounds and private cars (‘novel smoke-free policies’). An important element in the implementation of such policies is public support. In the context of the ambition of the Netherlands to reach a smoke-free generation by 2040, we investigated temporal changes in public support for novel smoke-free policies.

**METHODS:**

We analyzed annual cross-sectional questionnaires in a representative sample of the Dutch population from 2018 to 2022. Multivariable logistic regression was applied to model public support for each smoke-free policy area as a function of time (calendar year), smoking status, gender, and socioeconomic status. Interaction terms were added for time with smoking status and with socioeconomic status.

**RESULTS:**

A total of 5582 participant responses were included. Between 2018 and 2022, support increased most for smoke-free policies regarding train platforms (+16%), theme parks (+12%), beaches (+10%), and terraces (+10%). In 2022, average support was higher than 65% for all categories of smoke-free places and highest for private cars with children (91%). Regression analyses indicated significant increases in support over time within each category of smoke-free places (adjusted odds ratio, AOR between 1.09 and 1.17 per year), except smoke-free private cars with children (AOR=0.97; 95% CI: 0.89–1.05). Regardless of smoking status, support was high for places where children often go.

**CONCLUSIONS:**

Support for novel smoke-free places in the Netherlands is high and increasing, in particular for places frequented by children. This indicates the potential to implement such measures in the Netherlands.

## INTRODUCTION

To reduce exposure to secondhand tobacco smoke, the World Health Organization’s (WHO) Framework Convention on Tobacco Control (FCTC) urges countries to implement comprehensive smoke-free legislation in indoor workplaces, public transport and indoor public places^1^. A broad evidence base has identified clear health benefits of smoke-free policies across age ranges and settings^2-4^. Governments are now increasingly implementing smoke-free policies in outdoor public places or places that go beyond these recommendations. Examples are policies to regulate smoking at beaches, and in parks, playgrounds and private cars^5-8^, hereafter referred to as ‘novel smoke-free policies’^4,9^. Emerging studies indicate that such policies can successfully reduce secondhand smoke exposure, denormalize smoking^10,11^ and are associated with health benefits^4,12-14^.

The Netherlands is quickly moving up the European Tobacco Control Scale, from place 14 to 4 out of 37 between 2019 and 2021^15^. A smoke-free generation ambition was integrated into the National Prevention Agreement (NPA), a national plan involving the government and societal stakeholders to address tobacco use, problematic alcohol use, and obesity^16^. However, besides regulating smoking in outdoor areas of schools, no formal smoke-free policies were included in the NPA; they only shared ambitions with no legal basis.

Besides outdoor areas of schools and daycare centers, the NPA contained no other novel smoke-free policies. A timeline of key national tobacco control measures taken since 2008 is given in Table 1^17^.

**Table 1:** Timeline of key measures taken

*Year*	*Key measures*
2008	Ban on smoking in hotels, restaurants and cafes, tax increase and price increase of tobacco
2010	Ban on smoking in all cafes including those with no personnel other than the owner
2011	Smoking in small bars was allowed again
2013	Tax increase of €0.35 per 20-stick cigarette pack
2014	Increase of minimum age for buying tobacco from 16 to 18 years; smoking restriction again applied to small bars
2015	Tax increase of €0.09 per 20-stick cigarette pack
2016	Pictorial warnings on cigarette packs
2017	Ban on flavoured cigarettes except for menthol flavoured cigarettes
2018	National Prevention Agreement was signed, stating the ambition to reach a smoke-free generation by 2040
2020	All outside areas of schools smoke-free; display ban for tobacco products; €1.00 price increase per 20-stick cigarette pack; ban on menthol-flavoured cigarettes; implementation of plain packaging; train stations become smoke-free (implemented by the Dutch railway operators, not a governmental regulation)
2021	Ban on advertisement on the façade of a shop; ban on smoking rooms at government buildings and in public buildings
2022	Ban on cigarette vending machines; ban on smoking rooms at workplaces

For policymakers to consider implementing novel smoke-free policies, a comprehensive overview of trends in public support can be helpful. Previous literature has shown that data on public support can be used as a persuasive tool to increase the likelihood of policy implementation^18-20^. In the Netherlands, annual surveys on public support for various tobacco control measures have been conducted since 2009, showing that the Smoke-free Generation concept is well-known in Dutch society and appeals to most adults (73%)^21^. However, little is known about how support for novel smoke-free policies changed in this context. Therefore, this study investigates nationallevel trends in public support for novel smoke-free policies in the Netherlands from 2018 to 2022.

## METHODS

### Sample

We used data from a repeated cross-sectional annual survey from 2018 to 2022, by Kantar Public, a commercial institute for societal research, and commissioned by ‘Health Funds for a Smokefree Netherlands (GvRV)’. Each year, a sample of approximately 1600 Dutch adults were randomly invited from a panel, of which about 60–80% responded. The panel consists of respondents who have indicated that they are willing to participate in research regularly. Kantar Public actively recruited panel members, ensuring different subgroups of the Dutch population were represented. Each sample was drawn representatively for age, gender, education level, socioeconomic status (SES), and smoking status, and weighted to represent the Dutch population. For this study, potential participants were asked to complete a questionnaire concerning their opinion on several smoking-related questions. Respondents who participated in one year were excluded from participating in the next year, but could participate again the year after exclusion. Participants had to be at least 18 years old and Dutch-speaking.

### Participant characteristics

Participants were categorized according to gender (male or female), age in years (18–34, 35–54, ≥55), smoking status (non-smoker, ex-smoker, current smoker) and SES (low, middle, high). SES was based on a combination of the participant’s education and occupation (Supplementary file AI), where occupation consists of categories of job sector and management positions.

### Questionnaire

The surveys were conducted within two weeks in February 2020, 2021 and 2022, and between March and April 2018 and 2019. Participants were asked to answer, among other questions, twenty-two questions on support for smoke-free policies in several areas, which we categorized as follows: private cars with children, outdoor areas frequented by children (i.e. outdoor sports fields for children, petting farms, playgrounds, scouting areas, theme parks, and zoos), outdoor areas surrounding child care or educational facilities (i.e. 5–10 m surrounding daycare entrances, primary school grounds, secondary school grounds, and grounds of vocational education institutions, and of universities or universities of applied sciences), outdoor leisure areas (i.e. outdoor drinking and eating areas, beaches, soccer stadiums, outdoor swimming pools, parks, and outdoor sports fields), and areas surrounding buildings/public transport stops (i.e. in and around city halls, hospital grounds, and train station platforms).

Before 2018, support was assessed using the following question: ‘To what extent do you agree or disagree with a legal smoking ban in the following places? Completely agree, agree, neither agree nor disagree, disagree, completely disagree, don’t know.’. In 2018 and 2019, a pilot conducted by Kantar Public framed several questions, both supporting a smoking ban as well as a smoke-free zone in the same area. No significant differences were found between support for a ban and support for a smoke-free zone; therefore, positively framed questions were used when available in 2018 and 2019. From 2020 onwards, all questions were framed positively using the question: ‘Which of the following places should be completely smoke-free?’. In 2022, questions regarding outdoor educational facilities changed from ‘outdoor educational facilities’ to ‘outdoor and surrounding areas of educational facilities’. As this affects the comparability of the question to previous years, 2022 was not included for educational facilities.

### Analyses

Support for smoke-free locations was dichotomized into support (completely agree; agree) or no support (neither agree nor disagree; disagree; completely disagree; don’t know). Based on the most well-known determinants of public support^9^, multivariable logistic regressions were applied to model public support for each smoke-free policy area as a function of time (calendar year), gender, smoking status, and SES. Interaction terms were added between time and smoking status, and time and SES. Separate analyses were performed per category of smoke-free policies. As individuals could report support for several policies within each category, standard errors were clustered at the person level. For each analysis, weights were applied according to age, gender, SES, and smoking status to ensure representativeness of the Dutch society. Survey weights were calculated by Kantar Public. Analyses were conducted using R version 4.2.2, and a significance level of alpha=0.05 was assumed.

### Ethics

All respondents were part of Kantar Publics panel NIPObase and provided informed consent upon participation.

## RESULTS

In total, 5582 participant responses were included, varying between 988 in 2018 and 1358 in 2020. Table 2 presents the participant characteristics. Over time, the proportion of smokers decreased from 24% in the 2018 survey to 20% in the 2022 survey, reflecting the declining smoking rate in the Netherlands in this period.

**Table 2:** Characteristics of the unweighted sample, all years

*Characteristics*	*Overall n (%)*	*Smokers n (%)*	*Former smokers n (%)*	*Never smokers n (%)*
**Total,** n	5582	1191	2134	2257
**Gender**				
Male	2756 (49.4)	619 (52.0)	1132 (53.0)	1005 (44.5)
Female	2826 (50.6)	572 (48.0)	1002 (47.0)	1252 (55.5)
**Age** (years)				
18–34	1228 (22.0)	302 (25.4)	194 (9.1)	732 (32.4)
35–54	1892 (33.9)	456 (38.3)	559 (26.2)	877 (38.9)
≥55	2461 (44.1)	433 (36.4)	1381 (64.7)	647 (28.7)
**Socioeconomic status**				
High	2564 (46.0)	453 (38.1)	871 (40.8)	1240 (55.0)
Middle	1070 (19.2)	253 (21.3)	366 (17.2)	451 (20.0)
Low	1943 (34.8)	483 (40.6)	896 (42.0)	564 (25.0)
**Daily smokers**				
Yes		906 (76.1)		
No		285 (23.9)		
**Intention to quit**				
Yes, within one month		130 (10.9)		
Yes, within 6 months		235 (19.7)		
Yes, but not in the next 6 months		357 (30.0)		
No		469 (39.4)		

### Support

**Figure 1:**
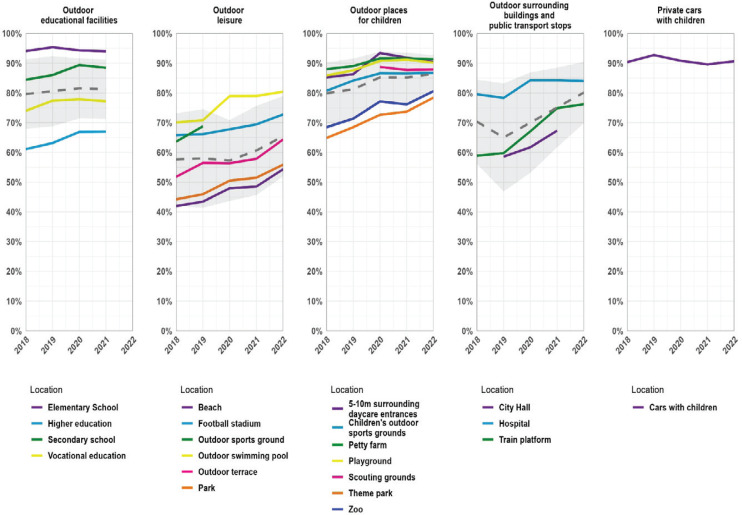
Public support for smoke-free policies, grouped by similar policies from 2018 to 2022. Dotted grey lines represent mean support per category and grey area represents the 95% confidence interval

In 2022, average support was higher than 65% for all categories of smoke-free places and highest for private indoor places (i.e. cars carrying children; 91%) (Figure 1). For each category of smoke-free policies, support increased between 2018 and 2022, with the largest increase (8 percentage points) for policies regarding outdoor areas surrounding buildings and public transport stops. Support for policies regarding outdoor places for children and private cars with children was consistently high across the study period (i.e. >90%). Within categories, support was highest for smoke-free elementary school grounds (93%, 2020 most recent), private cars with children (91%), petty farms (91%) and 5–10 m surrounding daycare entrances (90%). Concerning support for specific smoke-free locations, the largest percentage point increases between 2018 and 2022 were observed for train platforms (+16%), theme parks (+12%), beaches (+10%), and terraces (+10%).

### Determinants of support

Regression analyses indicated significant increases in support over time within each category of smoke-free places, except for smoke-free private cars with children, which was stable at around 90% (Table 3 and Supplementary file AII). Non-smokers and ex-smokers were more supportive compared to smokers for all types of policies. Absolute differences in support for smoke-free policies between smokers and non-smokers were especially large for outdoor leisure areas (∆48 percentage points in 2020) and for outdoor places surrounding buildings (Δ43 percentage points) and relatively small for private cars with children (Δ10 percentage points) (Supplementary file AII). Small differences between socioeconomic groups in support of smoke-free policies were observed; among those with a higher SES, support was generally higher, except for private cars with children (Table 3). Finally, compared to participants aged 18–34 years, people aged ≥55 years were more supportive of smoke-free policies. Interaction terms indicated no significant changes over time in relative support for any of the policies across categories of smoking status or SES.

**Table 3:** Determinants of support for novel smoke-free policies

	*Outdoor educational facilities*		*Outdoor leisure areas*		*Outdoor places for children*		*Outdoor surrounding buildings and public transport stops*		*Private cars with children*	
	*AOR*	*95% CI*	*AOR*	*95% CI*	*AOR*	*95% CI*	*AOR*	*95% CI*	*AOR*	*95 % CI*
**Variables**										
**Year**	**1.07**	**1.01–1.13**	**1.09**	**1.05–1.12**	**1.15**	**1.09–1.20**	**1.17**	**1.12–1.22**	**0.95**	**0.88–1.02**
**Smoking status**										
Non-smokers	1.00		1.00		1.00		1.00		1.00	
Ex-smokers	**0.61**	**0.52–0.71**	**0.61**	**0.55–0.68**	**0.67**	**0.57–0.78**	**0.59**	**0.51–0.67**	**0.86**	**0.67–1.11**
Smokers	**0.19**	**0.16–0.22**	**0.13**	**0.11–0.14**	**0.19**	**0.16–0.22**	**0.13**	**0.11–0.15**	**0.36**	**0.29–0.46**
**Gender**										
Female	1.00		1.00		1.00		1.00		1.00	
Male	0.97	0.86–1.09	0.93	0.85–1.02	0.91	0.81–1.02	1.06	0.95–1.18	0.81	0.67–0.98
**SES**										
High	1.00		1.00		1.00		1.00		1.00	
Middle	**0.80**	**0.68–0.93**	**0.80**	**0.71–0.90**	**0.74**	**0.63–0.87**	**0.74**	**0.64–0.85**	0.92	0.71–1.19
Low	**0.76**	**0.66–0.87**	**0.75**	**0.67–0.84**	**0.63**	**0.55–0.72**	**0.68**	**0.59–0.77**	0.85	0.67–1.07
**Age** (years)										
18–34	1.00		1.00		1.00		1.00		1.00	
35–54	**1.37**	**1.18–1.58**	1.03	0.91–1.17	1.05	0.90–1.23	**1.18**	**1.01–1.36**	0.94	0.74–1.20
≥55	**1.94**	**1.65–2.28**	**1.16**	**1.01–1.32**	**1.50**	**1.27–1.77**	**1.47**	**1.26–1.72**	**1.52**	**1.15–2.01**
Interactions*										
**Smoking status × Year**										
Non-smokers	1.00		1.00		1.00		1.00		1.00	
Ex-smokers	1.03	0.89–1.19	0.98	0.91–1.05	0.95	0.85–1.07	1.00	0.91–1.10	1.06	0.88–1.26
Smokers	0.98	0.85–1.12	0.94	0.86–1.04	0.90	0.80–1.01	0.92	0.83–1.03	0.85	0.71–1.01
**SES × Year**										
High	1.00		1.00		1.00		1.00		1.00	
Middle	1.07	0.92–1.24	1.01	0.92–1.11	0.94	0.83–1.07	1.03	0.92–1.15	1.05	0.86–1.27
Low	1.05	0.93–1.19	1.01	0.94–1.09	1.00	0.90–1.11	0.99	0.90–1.09	1.07	0.91–1.27

AOR: adjusted odds ratio; adjusted for all variables in the table. Statistically significant results in bold.

* Separate models per interaction term, corrected for calendar year, smoking status, gender, SES and age. SES: socioeconomic status.

## DISCUSSION

Between 2018 and 2022, repeated cross-sectional surveys among 5582 participants showed increasing or stable high levels of support for novel smoke-free policies in the Netherlands. While support was highest among non- and ex-smokers, it was 50% or higher among smokers for all places except outdoor leisure areas. In the general population, support was 50% or higher for all categories of smoke-free places. Regardless of smoking status, support was high for policies in places where children frequent, in particular for smoke-free cars when children are present.

Our findings indicate high levels of support for extending smoke-free policies in the Netherlands, particularly in places where children frequent. This corresponds with the smoke-free generation approach, which has become clearly embedded in Dutch society and national policy-making^17^. In line with this, support for smoke-free school grounds, for example, is higher as the age of the children that the educational institutions serve becomes lower: support is far over 90% for elementary schools, while for higher education, it is slightly below 80%. The patterns and levels of support in our study correspond to support levels found in a recent systematic review and meta-analysis of over 100 studies from 33 countries^22-25^, especially the high levels of support for smoke-free places where children frequent^9^. In line with findings from similar studies in other countries^9^, support for novel smoke-free policies in the Netherlands lagged behind in certain population subgroups, including people who smoke and those with low SES. Although support in these groups did not catch up over time, it was generally still quite high.

Despite high levels of support, the number of smoke-free policies beyond enclosed public places and workplaces is low in the Netherlands. Potential reasons might be lower belief in the effectiveness of smoke-free zones in these areas or challenges related to enforcement. Regarding the implementation of smoke-free policies in outdoor areas, an often-heard counterargument is the rationale that in well-ventilated areas, the possible health gains are limited. SHS exposure in well-ventilated places is, however, not insignificant, especially in outdoor places next to enclosed places with a smoking ban, such as offices or hospitals^26,27^. Enforcement in large open areas or in private areas indeed may be challenging. However, high levels of support can be an indicator of a norm change where smoking is no longer perceived as normal. Finally, an important argument supporting the implementation of smoke-free policies in these places is that reductions in SHS exposure and health benefits have been demonstrated despite existing challenges in enforcement^4,12,28-30^.

Future research is needed to better understand the determinants of public support for smoke-free policies in relation to the local political and cultural context. Also, more work is needed to see how public support may inform future policy-making. Additional studies identifying the impact of novel smoke-free policies on tobacco smoke exposure, smoking behavior, and health outcomes are also needed.

This is one of few studies assessing temporal trends in support of novel smoke-free policies in a large, nationally representative sample. As these data go up to 2022, it is possible that COVID-19 mitigation measures influenced support. While initially, support for the Dutch government was high in this period, this later on changed to much more distrust. Whether this dissatisfaction trickled down to support for smoke-free policies cannot be tested within our data but could be suggested for future research.

### Limitations

This study comes with several limitations. The results should be interpreted in the Dutch context and, therefore, might not be generalizable to other countries. Despite our attempts to control for confounding, residual confounding might be present. Furthermore, survey studies are at risk of bias. By using survey weights and representative sampling, we tried to minimize this risk. There are some limitations regarding measuring support. Within the study period, there were slight changes in the wording of the questions assessing support, from negatively framed (support for a smoking ban) to positively framed (support for a smoke-free area). However, no significant differences were found according to whether questions were asked in a negative or positive way, within the same questionnaire. Furthermore, there is no validated scale to measure support, and reliability is challenging to check. One might argue that support is hypothetical and does not reflect support for actual implemented policies. However, previous literature has shown that support often increases following implementation^23^. In our data, support following implementation of smoke-free areas surrounding elementary schools remained high following implementation in 2020^24,25^.

## CONCLUSIONS

Public support for novel smoke-free policies is relatively high and still increasing in the Netherlands, and for some policies has reached a plateau at a high level of support. This indicates substantial momentum for implementation, particularly for areas frequented by children, including cars carrying children.

## Data Availability

The data underlying this article were provided by Health Funds for a Smoke-free Netherlands. Data will be shared on request with the corresponding author with the permission of Health Funds for a Smokefree Netherlands.
