# Supplementary material for: Public support for smoke-free private indoor and public outdoor areas in the Netherlands: A trend analysis from 2018–2022
Source: Tob Induc Dis. 2024 Jan 17;22:10.18332/tid/176141. doi: 10.18332/tid/176141 (PMC10792619; doi:10.18332/tid/176141)
Supplement: Supplementary file 1 [file TID-22-12-s1.pdf]

## Appendix I

Education was divided into eight categories with an ordinal scale: ranging from no education to postdoctoral. Occupation was divided into 25 categories without an ordinal scale. The combination of the participant's education and occupation lead to the following possible outcomes with an ordinal scale: A, B1, B2, C, and D. Being categorised into group A meant having a prominent role, such as a researcher or a manager. Being categorised into group D meant having a basic profession or no profession at all. With everything else in between A and D. Subdividing these outcomes further, resulted in the following outcomes that ultimately were used in this research: High = A, B1, Mid = B2, Low = C, D.

| | | OPLGENOT_HKW | | | | | | | |
|---|---|---|---|---|---|---|---|---|---|
| | | Geen of basisonderwijs | LBO / VMBO (kader- of beroepsgericht) / MBO 1 / VBO | MAVO / HAVO of VWO (eerste drie jaar) / VMBO (theoretisch of gemengd) / (M)ULO | MBO 2, 3, 4 of MBO vóór 1998 | HAVO of VWO (4e, 5e of 6e klas) / HBS / MMS | HBO of universitair propedeuse | HBO of universitair bachelor/kandidaats | HBO of universitair master/doctoraal/postdoctoraal |
| BEROEPMOA1_HKW | Zelfstandig ondernemer | b2 | b1 | a | a | a | a | a | a |
| | Militaire beroepen Niet-Leidinggevend | c | c | b2 | b2 | b2 | b1 | b1 | b1 |
| | Militaire beroepen; Leidinggevend | c | c | c | b1 | b1 | b1 | b1 | a |
| | Managers; Niet-Leidinggevend | c | c | b2 | b1 | b1 | a | a | a |
| | Managers; Leidinggevend | b2 | b1 | a | a | a | a | a | a |
| | Onderzoekers,Ingenieurs,Docenten en specialisten; Niet-Leidinggevend | c | c | b2 | b2 | b1 | b1 | b1 | a |
| | Onderzoekers, Ingenieurs, Docenten en specialisten; Leidinggevend | c | c | b2 | b1 | b1 | a | a | a |
| | Vakspecialisten; Niet-Leidinggevend | c | c | b2 | b2 | b1 | b1 | b1 | a |
| | Vakspecialisten; Leidinggevend | c | c | b2 | b1 | b1 | a | a | a |
| | Administratief Personeel; Niet-Leidinggevend | c | c | c | b2 | b1 | b1 | b1 | a |
| | Administratief Personeel; Leidinggevend | c | c | b2 | b2 | b1 | b1 | b1 | a |
| | Dienstverlenend Personeel en Verkopers; Niet-Leidinggevend | c | c | b2 | b1 | b1 | b1 | b1 | a |
| | Dienstverlenend Personeel en Verkopers; Leidinggevend | c | c | b2 | b1 | b1 | b1 | b1 | a |
| | Landbouwers, Bosbouwers en vissers; Niet-Leidinggevend | c | c | c | b2 | b2 | b1 | b1 | b1 |
| | Landbouwers, Bosbouwers en vissers; Leidinggevend | c | c | c | b2 | b1 | b1 | b1 | b1 |
| | Ambachtslieden; Niet-Leidinggevend | c | c | c | b2 | b2 | b1 | b1 | b1 |
| | Ambachtslieden; Leidinggevend | c | c | c | b2 | b1 | b1 | b1 | b1 |
| | Bedieners Machines en Installaties, assemblagemedewerkers; Niet-Leidinggevend | c | c | c | b2 | b2 | b1 | b1 | b1 |
| | Bedieners Machines en Installaties, assemblagemedewerkers; Leidinggevend | c | c | c | b2 | b1 | b1 | b1 | b1 |
| | Elementaire Beroepen; Niet-Leidinggevend | d | d | c | c | b2 | b2 | b2 | b2 |
| | Elementaire Beroepen; Leidinggevend | c | c | c | b2 | b2 | b2 | b2 | b1 |
| | VUT/gepensioneerd | d | d | d | c | b2 | b1 | b1 | a |
| | Werkloos/bijstand/arbeidsongeschikt | d | d | d | c | c | b2 | b2 | b2 |
| | Studerend/Overig | d | d | d | d | c | b2 | b2 | b2 |
| | Onbekend | d | d | d | d | c | b2 | b2 | b2 |

source: https://moa04.artoo.nl/clou-moaweb-images/images/bestanden/pdf/samenvatting_Sociale_Klasse.pdf

**Appendix II**

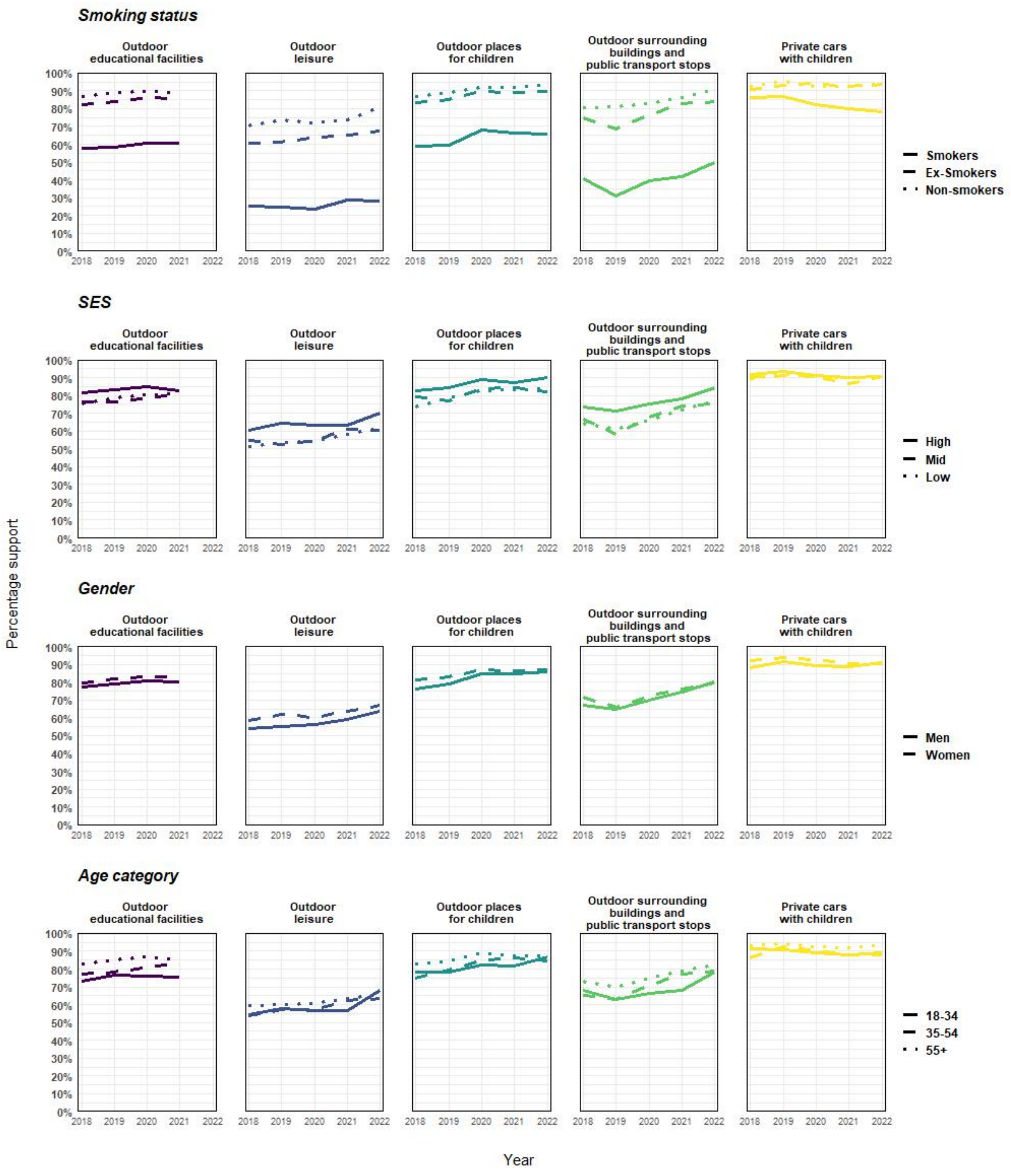


**Figure A1:** Percentage support by smoking status, SES, gender and age

© **2024 Boderie N.W. et al.**
